# Toward an Adapted Neurofeedback for Post-stroke Motor Rehabilitation: State of the Art and Perspectives

**DOI:** 10.3389/fnhum.2022.917909

**Published:** 2022-07-14

**Authors:** Salomé Le Franc, Gabriela Herrera Altamira, Maud Guillen, Simon Butet, Stéphanie Fleck, Anatole Lécuyer, Laurent Bougrain, Isabelle Bonan

**Affiliations:** ^1^Rehabilitation Medicine Unit, University Hospital of Rennes, Rennes, France; ^2^Hybrid Team, Inria, University of Rennes, Irisa, UMR CNRS 6074, Rennes, France; ^3^Université de Lorraine, CNRS, LORIA, Nancy, France; ^4^Neurology Unit, University Hospital of Rennes, Rennes, France; ^5^Empenn Unit U1228, Inserm, Inria, University of Rennes, Irisa, UMR CNRS 6074, Rennes, France; ^6^EA7312 Laboratoire de Psychologie Ergonomique et Sociale pour l’Expérience Utilisateurs (PERSEUS), Metz, France

**Keywords:** brain–computer interface, neurofeedback, stroke, motor rehabilitation, brain plasticity

## Abstract

Stroke is a severe health issue, and motor recovery after stroke remains an important challenge in the rehabilitation field. Neurofeedback (NFB), as part of a brain–computer interface, is a technique for modulating brain activity using on-line feedback that has proved to be useful in motor rehabilitation for the chronic stroke population in addition to traditional therapies. Nevertheless, its use and applications in the field still leave unresolved questions. The brain pathophysiological mechanisms after stroke remain partly unknown, and the possibilities for intervention on these mechanisms to promote cerebral plasticity are limited in clinical practice. In NFB motor rehabilitation, the aim is to adapt the therapy to the patient’s clinical context using brain imaging, considering the time after stroke, the localization of brain lesions, and their clinical impact, while taking into account currently used biomarkers and technical limitations. These modern techniques also allow a better understanding of the physiopathology and neuroplasticity of the brain after stroke. We conducted a narrative literature review of studies using NFB for post-stroke motor rehabilitation. The main goal was to decompose all the elements that can be modified in NFB therapies, which can lead to their adaptation according to the patient’s context and according to the current technological limits. Adaptation and individualization of care could derive from this analysis to better meet the patients’ needs. We focused on and highlighted the various clinical and technological components considering the most recent experiments. The second goal was to propose general recommendations and enhance the limits and perspectives to improve our general knowledge in the field and allow clinical applications. We highlighted the multidisciplinary approach of this work by combining engineering abilities and medical experience. Engineering development is essential for the available technological tools and aims to increase neuroscience knowledge in the NFB topic. This technological development was born out of the real clinical need to provide complementary therapeutic solutions to a public health problem, considering the actual clinical context of the post-stroke patient and the practical limits resulting from it.

## Introduction

Stroke is a major public health problem. In 2010, about 17 million incident stroke cases were responsible for more than 3 million deaths worldwide (Feigin et al., 2014). It is a leading cause of severe acquired disability in adults in industrialized countries (Lecoffre et al., 2017). More than 60% of the stroke subjects present severe and persistent upper limb motor injury without a useful grip (Nakayama et al., 1994). Furthermore, it decreases the subjects’ autonomy and activities of daily living, so motor recovery constitutes a major rehabilitation issue (Broussy et al., 2019).

Recent research progress on brain plasticity and development of technologies have led to various therapeutic proposals for post-stroke motor rehabilitation. The patho-neurophysiological post-stroke changes of motor recovery occur mainly within the first 15 weeks after the event, regardless of the severity of the initial motor deficit (Stinear, 2017). From 6 months onward, the motor deficit is considered stable and chronic (Langhorne et al., 2011). However, there is evidence that motor function continues to improve in the chronic phase through different cerebral plastic reorganization mechanisms that complement each other (Di Pino et al., 2014). Therefore, the development of new rehabilitation tools is a major goal in the healthcare field regarding post-stroke recovery in addition to traditional therapies. The major challenge is to use these new treatments in a way to better meet the patient’s needs: taking into account their deficiencies (motor and other), the type of injury, the time since the stroke, and their abilities. The ultimate goal is to use the best therapeutics to meet the patients’ needs when they can be offered. Over the past decade, studies have revealed the potential of Brain–Computer Interfaces (BCI), including Neurofeedback (NFB), to stimulate neural plasticity in motor areas of the brain and promote functional improvement (Sreedharan et al., 2013). There are many rehabilitation techniques used and recognized in conventional therapies for stroke rehabilitation, which can be associated with BCI technologies. They include robotic technologies, non-invasive brain stimulation, mirror therapy, or action-observation (Faralli et al., 2013; Belagaje, 2017; Raffin and Hummel, 2018).

**FIGURE 1:**
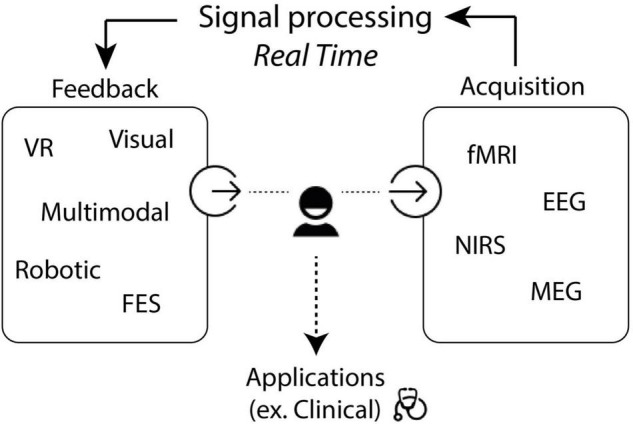
Description of the Neurofeedback loop. NFB, neurofeedback; EEG, electroencephalography; fMRI, functional magnetic resonance imaging; NIRS, near infrared spectroscopy; MEG, magnetoencephalography.

Neurofeedback therapy using a BCI allows a closed-loop system that provides real-time information to the participant regarding his/her brain activity, which can be used to develop self-learning strategies to modulate one’s brain signals. The brain physiological parameter related to the function to be improved or the brain biomarker, such as brain activity in motor areas for motor recovery, is measured and processed by a technological interface to provide the participant with simple, continuous, and real-time information to allow self-regulation. The triggering of the intended biomarker is rewarded and positively reinforced with feedback that could motivate the subject and support learning (Figure 1; Wolpaw et al., 2020). This technology has been used for motor post-stroke rehabilitation highlighting interesting results when combined with traditional therapies, in brain changes, reducing maladaptive plasticity, enhancing ipsilateral primary motor cortex activity, and clinical recovery based on clinical scales (Carvalho et al., 2019; Bai et al., 2020). Although NFB for post-stroke recovery showed encouraging results, it remains an emerging technique in clinical practice with neurophysio-pathological and clinical issues. Many NFB studies have already been performed, but with various neuroscientific, technical approaches and heterogeneous protocols. The clinical profiles of the stroke subjects, the patterns of brain lesions, the time elapsed after stroke, the clinical program of NFB therapy, or the devices used are part of the criteria that can influence the results of the studies, with challenges. The recent literature even concluded that more homogenous studies were needed to better understand the brain changes brought by the NFB (Baniqued et al., 2021; Yoo, 2021). On the other hand, several authors claimed the necessity to adapt the NFB to each profile of the participants (Kruse et al., 2020).

Considering all these remarks, we aimed to propose criteria allowing an adjustment of each parameter according to the subject’s clinical situation, i.e., according to their injury, deficiencies, and the device’s technical limitations to maximize recovery of motor functions. Thus, we could propose an adaptation of the NFB procedure.

Adapting therapeutic solutions is based on the principle of taking into account the heterogeneity of patients. Solution differentiation could correspond to one process of considering heterogeneity within a population. It might propose different solutions to groups of individuals according to typologies that gather them into subgroups with distinct needs. The differentiation of solutions could then go as far as individualization or even personalization (Metz et al., 2012; Bíró et al., 2018). The definition of individualization takes into account the individual characteristics of each person to best respond to their individual and specific needs. On the other hand, one could define the personalization taking the principle even further by considering not only the person’s needs but also their values, norms, passions, identity, etc., concisely, what defines them as unique, to make the solution their own. This process generally requires the person’s active participation, for example, via co-design. These two terms must not be confused in the literature.

The term of adaptation means the process of changing to better suit a situation. The adaptation process can lead to an individualization of care, considering the individual characteristics to best meet specific needs of a population. NFB adaptation could allow a more individual rehabilitation program tailored to the stroke subject’s situation to facilitate a more effective motor recovery.

In this work, we compiled the different NFB articles and collected from the literature the principal criteria to be studied in future research using a transdisciplinary approach. First, we focus from a human centered perspective on the neuroscientific and clinical considerations: stroke clinical context, time since stroke, anatomical targets, motor imagery methods, and technological limitations. Then, from a techno-centered perspective, we describe various devices and their technological limits: explanation of the different acquisition systems, signal processing, and the feedback modalities involved.

## Methodology

We produced a non-systematic literature review of studies with a multidisciplinary approach. A computerized search was conducted using the following databases: PubMed, Web of Science, and Google Scholar. In each database, we searched using the combination of the following keywords “(Brain–Computer Interface OR Neurofeedback) AND (Stroke OR Cerebral Stroke).” We also conducted a manual search, including studies focused on the upper limb. There was no limitation date for the published articles; the last search was on 4 September 2021. We focused on BCI studies for post-stroke motor rehabilitation focused on cerebral plasticity modulation and inducing neuroplasticity. We did not consider the entire field of BCI technologies as it comprises a wide range of applications other than medical such as neuroergonomics, neuromarketing, entertainment, education, and security. Furthermore, the rehabilitation field here concerns the upper and also the lower limb, even if the literature of NFB is more widely developed for the upper limb.

### Toward a Neurofeedback Adapted to the Neuroscientific and Clinical Considerations

The clinical context after stroke is the central point of decision making for choosing the criteria to offer an optimal therapy to each individual with stroke. Therefore, in this section, we exposed, based on the literature, all the criteria to consider regarding the patient and the stroke event to adapt the NFB therapies to their needs as soon as possible. In that way, we focused on the adaptation of NFB depending on the time since stroke, the anatomical injured brain areas, the mental imagery (MI) methods, the ability to perform BCI tasks for subjects and the clinical context.

#### Time-Based Target

**FIGURE 2:**
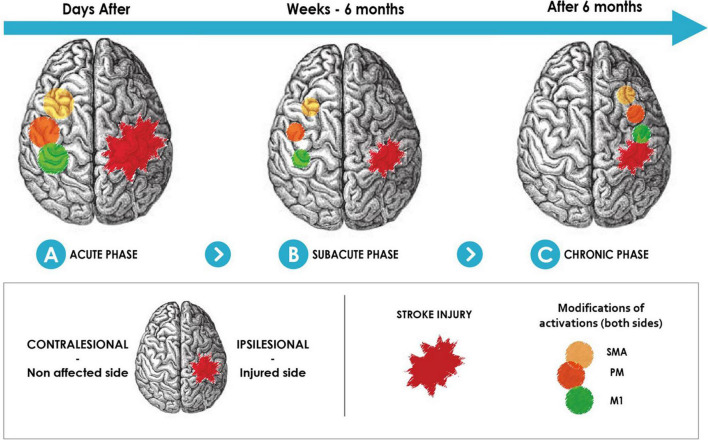
Physiological recovery mechanism in brain motor areas after stroke. Increasing activity of the non-affected side motor-related areas at the acute recovery phase **(A)**. Non-affected side brain motor area activity decreased in the first months after stroke onset **(B)**. Brain motor area activity in the affected side increases after 6 months **(C)**. SMA, supplementary motor area; PM, premotor area; M1, primary motor area.

Cerebral plasticity is known to be time-dependent due to the brain physiopathological mechanisms involved in the recovery evolution over time (Stinear, 2017). The time when the NFB is proposed is therefore of great importance. During the first months, changes in ipsilesional and contralesional motor cortical excitability are essential and demonstrate the importance of brain plasticity processes (Figure 2). Some studies showed the role of ipsilesional motor areas at the early stage after stroke (Volz et al., 2016; Volz, 2017).

**FIGURE 3:**
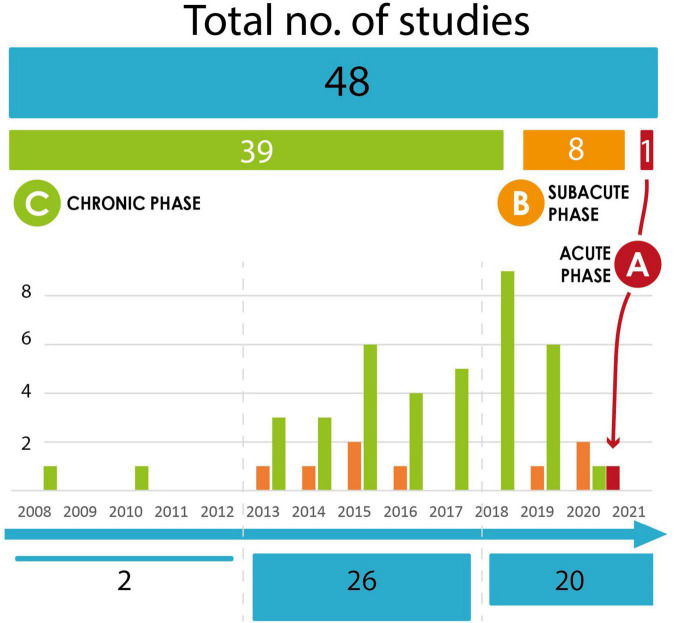
Evolution of NFB studies in the literature. Number of NFB studies in stroke population according to the time since the stroke. The studies included in the graph are those cited in the main meta-analyses since 2018 (Carvalho et al., 2019; Bai et al., 2020; Kruse et al., 2020; Baniqued et al., 2021). **(A)** Acute phase. **(B)** Subacute phase. **(C)** Chronic phase.

Six months is considered the period beyond which as the time that changes caused by cerebral plasticity become stable and spontaneously non-evolving (Langhorne et al., 2011; Di Pino et al., 2014). Motor-training NFB has been widely used in the chronic post-stroke population (after 6 months). Numerous systematic reviews and meta-analyses concerning NFB have shown interesting results about motor recovery in the short and long term in the chronic post-stroke population (Carvalho et al., 2019; Bai et al., 2020; Baniqued et al., 2021) (Figure 3). Bai’s meta analysis described 13 controlled randomized studies. Among the feedback used, two studies used visual feedback, six studies used orthosis, four studies tested functional electrical stimulation (FES), one study compared visual versus orthosis feedback. The control groups used feedback without NFB, or traditional rehabilitation NFB training lasted between 10 and 30 sessions, targeting mu rhythms. Clinical upper limb motor function improved significantly in the NFB group compared to the control group. The advantage in this chronic stage is therefore to evaluate the cerebral changes in a stable situation.

Rare studies focused on the acute (<7 days) or subacute (until 6 months) post-stroke stages, when brain changes evolve quickly. At this delay after stroke, NFB protocols involve delicate choices of anatomical targets, considering the cortical brain changes, the fatigue and general state of the subject, as well as the logistical difficulties of feasibility studies at the patient’s bedside. One feasibility study evaluated the safety of motor EEG-NFB training as early as seven days after stroke and found positive results (Hashimoto et al., 2021). They tested in four inpatients with moderate to severe upper limb disability a head-mounted display with wrist electrical stimulation as feedback based on their MI performance, safely following the protocol at the bedside. Wu’s study showed the relevance when using EEG-based BCI motor training triggering mu rhythms. They used a combined visual and orthosis feedback in the NFB protocol with a control group without NFB. Patients were included between 1 and 6 months after stroke. They demonstrated brain connectivity changes and better clinical improvement in the BCI group compared to the control group in 25 participants after stroke following a 4 weeks-training in a controlled randomized study (Wu et al., 2020). In Pichiorri’s study, they also performed a controlled randomized study and they included 28 subjects from 6 weeks to 6 months after stroke with severe motor impairment. They found a better clinical improvement in the NFB group with visual feedback compared to the MI group (Pichiorri et al., 2015). Another ongoing study includes the same population from 1 to 6 months after stroke to evaluate the short and long-term efficacy of NFB training (Mattia et al., 2020). They use visual ecological feedback with the participants’ own hands. The large time interval since the stroke among the patients and the difference in brain plasticity process at each stage limited the conclusion in all the studies. More recently, one meta-analysis focused on comparing the efficacy of NFB studies in subacute (four studies) and chronic (eight studies) stroke participants (Mansour et al., 2021). Although the effect size was in favor of NFB interventions in both groups, it was more important in the subacute group taking into account its population heterogeneity. Nevertheless, it is difficult not to consider the spontaneous recovery of the subjects before 6 months, knowing the physiological brain plasticity.

Therefore, it is necessary to better understand the physiopathological mechanisms concerning the cerebral plasticity over time since the stroke to optimize the NFB training according to the post-stroke period (Cramer, 2008; Hara, 2015) and, in particular, the implication of the non-affected side motor-related area cortex activity over stroke phases (Figure 2). The limits here would be to stimulate plasticity in an inappropriate way at the acute, subacute, and even chronic stage because the protocols are not adapted to the ongoing cerebral plasticity (Murase et al., 2004; Boddington and Reynolds, 2017). In addition, the medical and general state (fatigue, concurrent medical problem, among others) must also be taken into account to design the protocols and understand the obstacles during their development and are often part of the exclusion criteria in the protocols.

To summarize, the adaptation of the NFB should consider the delay since the stroke. For this purpose, more knowledge is needed about the natural evolution of cerebral plasticity according to the subjects’ clinical profiles. Additionally, the challenge is probably to practice NFB-based rehabilitation early after the stroke to increase motor recovery. Finally, the homogenization of the cohort of patients considering the stage after stroke is necessary to deliver relevant results.

#### Choice of Brain Biomarkers for Enhancing Motor Recovery

Besides the intervention time of the rehabilitation therapy, the cerebral anatomical area and, more generally, the brain network to stimulate, remains a question. To the best of our knowledge, most studies have estimated the impact of NFB by stimulating the ipsilesional primary motor cortex (M1) (Figure 2) and the associated premotor areas in motor post-stroke rehabilitation. This approach is based on functional brain imaging data exploring neuroplasticity (Favre et al., 2014). They showed that the increase in activations observed days after stroke was rather contralesional with a decrease in activation in the injured cortex. In case of good recovery, a re-lateralization of activations can be observed in the ipsilesional motor areas in the following weeks. The persistent use of the compensatory contralesional network results in a poor-quality recovery in the hemiplegic adult. This cerebral plasticity using the contralesional hemisphere can be considered maladaptive and non-optimal, probably because the cortico-spinal pathway is too damaged. NFB protocols have focused on reactivating damaged motor areas and the adjacent cerebral motor and premotor zones from this reasoning. These stimulation areas, although encouraging, do not have optimal results.

Therefore, other ipsilesional targets have been considered. The supplementary motor area (SMA) is an easily activated location during NFB-based training (Mehler et al., 2019). SMA is a cerebral area vascularized by the anterior cerebral artery and is often spared during ischemic damage to the primary motor cortex, vascularized by the middle cerebral artery, and directly related to the corticospinal tract (CST). However, SMA’s real implication in motor recovery and its use is not well understood. It was tested in the motor rehabilitation post-stroke for the upper limb in one proof-of-concept study (Mehler, 2020). NFB with functional magnetic resonance (fMRI) was provided from the SMA targeting two different NFB target levels (low and high). The analysis found that the stroke subjects struggled to activate the SMA during motor imagery in NFB training. A controlled trial tested the SMA as a target with functional near-infrared spectroscopy mediated NFB (fNIRS-NFB) (Mihara et al., 2021). The goal was to increase post-stroke gait and balance recovery in subjects with subcortical stroke. The primary outcome was the 3-m-Timed Up-and-Go (TUG) test improvement, 4 weeks post-intervention. The results showed a greater improvement in the NFB group compared to the control group. It seems, from this preliminary data, that the SMA may not be used in rehabilitation for all motor abilities. Similarly, other targets may be considered for NFB like the ipsilesional left premotor cortex (Marins et al., 2015) or premotor areas (Plow et al., 2015).

Nevertheless, the stroke lesions are not similar from one person to another, and the motor circuit’s alteration is different. Thus, it is plausible that the areas to be stimulated for motor recovery need to be adapted according to the characteristics of the injury. The influence of the lesion characteristics, whether in terms of size or location, on the clinical effect of NFB is rarely analyzed in studies. In a meta-analysis (Carvalho et al., 2019), it was underlined that most participants had a subcortical stroke with thus a supposed better MI capacity. The recovery of hand function following lesions in the primary motor cortex is associated with a reorganization of premotor areas in the ipsilesional hemisphere. This reorganization seems depend on the size of the lesion (Touvykine et al., 2016).

Additionally, neuroimaging and electrophysiology markers exploring the damage to the anatomical structure as the CST predict motor recovery for the upper limb (Stinear, 2017) or for walking (Soulard et al., 2020), which measures the integrity of the CST and could be used to test the effectiveness of NFB for upper limb motor recovery. Apart from these large-scale anatomical observations, motor functions are complex. They go through different neural circuits and are integrated into networks. Therefore, it is essential to understand the impact of the lesion on this loop, and its physiopathological implication post-stroke to improve the rehabilitation target. For example, shoulder muscles are innervated bi-hemispherically, whereas hand muscles are mostly innervated contralaterally (Carson, 2005). Reproducing this spatial specificity activation in EEG-NFB is possible (Hayashi, 2020) but has not been tested in stroke subjects.

When a lesion is extensive, and the area would not be “recoverable,” it is possible to activate other circuits than the principal motor network. This approach was considered in a non-invasive stimulation context, with the cerebellum as the target (Wessel and Hummel, 2018). From this same concept, it could be considered to stimulate in a “weighted” way the various circuits according to the affected areas, and according to the time after the stroke. Thus, accessory networks can play a role in neuroplasticity when primary networks are too injured and could be a future work target (Jang et al., 2013; Jang and Cho, 2022).

Considering that motor recovery is done within the network, new biomarkers such as connectivity are emerging. They take into account the brain’s ability to communicate at a distance and functionally interact in a network (Mottaz et al., 2015; Gonzalez-Astudillo et al., 2021). One double-blind controlled study used it in post-stroke motor rehabilitation with a significant clinical result (Mottaz et al., 2018).

Anatomical targets of the NFB may be more precisely adapted. More investigations are needed to understand the clinical impact of NFB in the adjacent brain areas in rehabilitation, adapt the target to be stimulated according to the lesion, and the clinical impact sought. The main obstacle is the spatial specificity limited by the acquisition system used. It is currently only feasible to target specific areas with advanced technology such as MRI. The use of NFB-MRI is limited in current clinical practice. Thus, targeting specific areas in NFB protocols remains experimental.

The insufficient current knowledge of the diverse physiopathological mechanisms of motor recovery after stroke according to the lesion sites to be rehabilitated limits our ability to target the appropriate motor areas or network in NFB. Therefore, a future challenge would be to evaluate the integrity of the different networks and use it as targets in NFB, taking into consideration the healthy areas and the possibilities of vicariance, thus adapting the rehabilitation to the clinical recovery sought.

#### Motor Imagery or Motor Attempt

Motor Imagery (MI) is the basic tool to stimulate motor brain areas in EEG-based NFB. MI activates brain structures that share similar neural networks with motor execution, including pre-motor, supplementary motor, cingulate, parietal cortical, basal ganglia, and cerebellum areas (Decety, 1996; Kilintari, 2016). A recent meta-analysis (Monteiro et al., 2021) concluded that MI, as a complementary resource to traditional rehabilitation in post-stroke subjects, is effective to improve motor function and functional dependence. There are two types of MI: visual motor imagery (VMI) and kinesthetic motor imagery (KMI). The latter consists of a mental process which can be described as the ability to imagine performing a movement without executing it, specifically by reactivating the haptic sensations (i.e., tactile, proprioceptive, and kinesthetic) felt during a real movement and seems more appropriate in NFB (Guillot et al., 2008). Although more difficult to develop than the VMI, it allows the same neural networks to be activated as real movements in functional imagery (Chholak et al., 2019), and therefore seems to be preferred in the field of rehabilitation (Halder et al., 2011). This concept is close to the motor attempt (MA) consisting of performing the intention of movement. Mansour’s meta-analysis found interesting results considering motor function recovery with superiority of MA compared to MI in a post-stroke population (Mansour et al., 2021).

Motor imagery can be used differently depending on the purpose. In NFB, MI is considered the trigger of cerebral plasticity, detecting neuronal modulation that is supposed to help stimulate the motor brain targets. More broadly in BCI, the feedback helps to modulate MI productions (Padfield et al., 2019).

To implement NFB, we must consider the ability of the participants after the stroke to perform MI or MA. Several questionnaires such as the Kinesthetic and Visual Imagery Questionnaire (KVIQ) (Malouin et al., 2007) and the Motor Imagery Questionnaire-Revised Second Edition (MIQ-RS) (Loison et al., 2013) have been employed in the literature to evaluate the preference and the capacity of healthy subjects and post-stroke subjects to perform MI. The KVIQ scores, which determine the subjects’ ability to feel and visualize the imagined movement, have been strongly correlated to offline BCI performances for the classification of left versus right hand MI (Vuckovic and Osuagwu, 2013). Nevertheless, these questionnaires present some limitations. Some studies made a point about the reliability of these tools. Pillette’s study did not find any influence of the initial KVIQ scores on MI performances (Pillette et al., 2021). Concerning MIQ-RS, no significant correlation was found between the scores and the BCI performance in healthy subjects (Rimbert et al., 2019). Thus, the results from the questionnaires must be taken cautiously. Another study showed that cognitive patterns could influence the capacity to perform MI, mainly the visuospatial performance and abstraction skills (Jeunet et al., 2015a). Our understanding of sources of variability in motor imagery performance between individuals remains incomplete. Developing new questionnaires with better correlation between scores and performance could be interesting. Moreover, some studies demonstrated the benefit of doing a motivating and relevant MI task for the subject instead of a specific repeated movement (Strehl, 2014). In addition, interesting results in healthy subjects showed that focusing on feedback (e.g., the feedback gauge) or focusing on MI strategy was not in favor of good performance for some people, considering that no specific task would be preferable and could lead to better performance for some individuals (Kober et al., 2013). This suggests that the more spontaneous and natural the MI task is for the subject, the more effective it will be.

The MI technique is mainly studied in healthy subjects and is often unspecified in the literature. Therefore, there are many ways to practice MI or MA, depending on the participant’s skills, preferences, performances, and purpose. In practice, MI remains uncontrollable.

There are few data and tools in the literature to measure the effectiveness of MI and little is yet known about its action mechanism. The data acquisition of the brain activity might be adapted to the expected activity evoked by the MI properties for the different clinical contexts of the post-stroke population.

#### User’s Disability in Brain–Computer Interfaces

A concept called “BCI illiteracy” emerged in the literature a few years ago concerning the ability of subjects to successfully perform BCI protocols. It is estimated from 15 to 30% of the population cannot successfully control BCIs (Allison and Neuper, 2010) and may contribute to the limits of the feasibility of NFB studies. Some authors have shown that performance variation in BCI could depend on certain profiles (Ahn and Jun, 2015). In this review based on healthy subjects, they determined that psychological, anatomical, and physiological patterns could influence the ability to practice BCI. For example, music players were considered good BCI performers (Randolph, 2012). Moreover, fMRI studies in healthy participants have found that higher activation of SMA occurred for good BCI performers (Halder et al., 2011) and parietal and premotor brain areas were also more activated by them (Guillot et al., 2008). Once again, these results come from the literature on healthy subjects. We can imagine the additional difficulty when brain lesions happen for subjects who try to perform a BCI protocol. Cognitive, visuospatial, or motor lesions may worsen the difficulties to practice NFB and MI. Thus, depending on the individual stroke subjects’ profiles and the injured brain structures after stroke, the NFB protocol may be ineffective due to user inability, with a risk of lower performance.

Although some users may present some difficulties to perform BCI, it should not be forgotten that the changes of paradigm, biomarker or even the adaptation of feedback and training modalities can allow an adequate performance. We must evaluate the participants ability to practice BCI tasks to better adapt the therapy to their needs, also considering that the MI performance is linked to the BCI ability.

#### Clinical Consideration

The clinical context should be considered in post-stroke NFB protocols. Patients may have symptoms that limit their access to this type of rehabilitation. Considering that these NFB therapies can be offered when other rehabilitation treatments are not sufficient, it means that the patient’s clinical condition is more severe (upper and/or lower limb motor deficit, sensitive deficit, spasticity). Spasticity is a common symptom in 50% of stroke patients, and is often correlated with the severity of the motor deficit (Opheim et al., 2015). However, spasticity can interfere with the NFB protocol, particularly in the use of the interfaces and feedbacks used (see section “General Recommendations for an adapted Neurofeedback for stroke”). The localization of stroke and the other impairments as cognitive and emotional dysfunction can also lead to difficulties for patients to follow a rehabilitation that will not necessarily take all these deficiencies into account. In a recent review, the authors discussed the interest to target the regulation of cognitive and emotional disorders in NFB to help the clinical improvement (Young et al., 2014; Nan et al., 2019; Mane et al., 2020). Indeed, it is important to take into account symptoms such as apathy, depression, fatigue, concentration impairments which are very frequent after a stroke and which can disrupt access to rehabilitation (Robinson and Jorge, 2016; Li et al., 2019).

### Toward an Adapted Technological System for Post-stroke Motor Rehabilitation

The previous section highlighted the importance of considering the singularity of the patients to improve the NFB training program with a better human-centered approach. At the same time, the diversity of existing NFB technological solutions increases. Therefore, it also seems essential to engage in a techno-centered reflection to cover the field of possibilities. For this purpose, we presented the specialized technological systems that compose NFB therapies by separating them according to their functionality: acquisition system, signal processing, and feedback modalities. First, an acquisition system is needed to record brain activity. Non-invasive systems include electroencephalography (EEG), functional near-infrared spectroscopy (fNIRS), functional magnetic-resonance-imaging (fMRI), and magnetoencephalography (MEG). Once the brain activity has been measured, it must be processed to extract the relevant characteristics related to the brain biomarker. Finally, feedback based on the biomarker may be delivered in various possible forms, such as visually, haptically, or using multiple senses.

The two most studied systems in the literature are EEG and functional MRI. Nevertheless, there are intermediate systems in spatial and temporal resolution like fNIRS, with transportability advantages (Mihara and Miyai, 2016; Kohl et al., 2020). The primary technical goal of NFB research is to obtain the highest real-time information from brain activity in the most convenient and unobtrusive way, with the least setup time and calibration. The main persistent obstacle of these systems for their use in clinical practice remains their practicability in terms of equipment, technical mastery by a clinician (and the need for a trained patient to use the equipment), and its financial cost. Another limitation is the anatomical target reached depending on the acquisition system.

#### Acquisition System

The question of the acquisition system is important because of its impact on clinical practice and access to the brain structures. Currently, there are various non-invasive methods, including EEG, fNIRS, fMRI, and MEG, each with its advantages and limitations (Table 1). All these systems have been tried for NFB in a post-stroke context. Some tools, such as EEG, have been particularly developed because they are older technology and are easy to use in subjects with different clinical deficits. We will not mention invasive techniques as they are not very suitable for routine clinical practice yet. Instead, we focused more on EEG and MRI techniques which are more developed in the literature.

**TABLE 1:** Summary of acquisition systems and their specificities used in NFB studies in post-stroke context.

	EEG	fMRI	fNIRS	MEG
Principle	Measures the differences in electric potential between electrodes on the scalp.	Measures the blood-oxygen-level-dependent (BOLD) contrast: a change in magnetization between oxygen-rich and oxygen-poor blood correlated with neuronal activity.	Measures changes in the infrared light absorption between oxy-hemoglobin and deoxyhemoglobin from blood vessels on the surface of the brain.	Captures the magnetic fields generated by the movements of ions induced by the activity of neurons.
Biomarkers	ERD/ERS Connectivity	BOLD in the ROI Connectivity	HbO value Mean change HbO and HbR	ERD/ERS Connectivity
Spatial resolution (Olivi, 2011)	20 mm	1 mm	10 mm	15 mm
Temporal resolution (Olivi, 2011)	1 ms	1 s	<1 s	1 ms
Limitations	No	Metalic implantable medical devices Cochlear implants Claustrophobia	No	No
Targets	Cortical	Cortical or deep	Cortical	Cortical
Estimated installation time	About 15–20 min Depends on the number of electrodes Less if dry electrodes	1–10 min Depends on the motor deficit	1–5 min	1–5 min Depends on the motor deficit
Tolerability	++++	++	++++	+++
Side effects	No	No	No	No
Portability	Yes	No	Yes	No
Use in clinical practice	++++	++	+	+
Technical difficulties	Artifacts, movement	Artifacts, movement	Artifacts, movement	Artifacts, movement
Technical mastery		Requires trained personnel		Requires trained personnel
Equipment cost	100 – 20,000 euros	1–5 million + 300 euros per hour	15,000 euros	2 millions

*ERD, event-related desynchronization; ERS, event-related synchronization; HbO, oxygenated hemoglobin; HbR, deoxygenated hemoglobin; ROI, region of interest. Coupling of numerical methods for the forward problem in Magneto- and Electro-Encephalography (Olivi, 2011). +, low; ++, moderate; +++, high; ++++, very high.*

##### Electroencephalography

The EEG is the oldest tool for signal acquisition in NFB especially in the post-stroke context for upper limb rehabilitation. It allows the detection of pre-movement event-related desynchronization (ERD) and post-movement event-related synchronization (ERS) (Pfurtscheller et al., 1998) of mu and central beta rhythms, from electrodes placed on the scalp above the motor areas. The recorded brain signals are filtered and processed with specialized algorithms to obtain relevant information related to the movement.

EEG is the most used acquisition system in clinical practice (Bai et al., 2020), because of its greater ease of practicability. There are no associated contraindications, and its use is well tolerated. Several modern systems offer a larger number of electrodes with the possibility of high-resolution EEG and better spatial resolution. However, more electrodes is not always an advantage, specially in the clinical context where the installation time required for a large quantity of electrodes may represent a burden. This acquisition system is transportable to the patient’s bed or even to their home, but at the risk of a decreased performance (Nishimoto et al., 2018).

Nevertheless, EEG has limitations: many artifacts, limited spatial resolution, and activity from deeper structures is hardly observable. Moreover, it also requires time for the installation and a hair wash for the gel systems, which adds a burden to the patients. New research to improve EEG acquisitions and make them more accessible post-stroke practice is turning to the use of dry electrodes (Grozea et al., 2011; Pei et al., 2018). This allows for increased accessibility and comfort. In the same way, the use of more targeted areas would allow a selection of electrodes and a less heavy and simpler acquisition system.

##### Others

Other non-invasive systems have various points of interest for the spatial or temporal resolution (Table 1). Nevertheless, there are intermediate systems in spatial and temporal resolution like fNIRS, and with transportability advantages that are under development (Mihara and Miyai, 2016; Kohl et al., 2020). The main persistent obstacle of these systems for their use in clinical practice remains their practicality in terms of equipment, technical mastery by a clinician (and the need for a trained person to use the equipment), and their financial cost. Another limitation is the anatomical target reached depending on the acquisition system.

Recently, there has been a growing interest in combining more than one brain activity measurement technology, named hybrid system. The idea is to bring together different recording techniques that may be complementary to give back a more performant NFB. However, the application is challenging because two very different systems need to work together, synchronized, and without compromising real-time performance.

The principal hybrid system is an EEG-fMRI-NFB. The EEG system with a good temporal resolution allows it to complement the fMRI-NFB with a better spatial resolution. This approach enables the complementarity of these two modalities (Mano and Barillot, 2017; Perronnet et al., 2018). A pilot study used it in clinical practice for the motor rehabilitation of limb with chronic stroke participants (Lioi et al., 2020). Preliminary results suggested its feasibility, and more importantly, this study was a relevant way to understand the mechanisms and limits of NFB: only two subjects with a preserved CST and subcortical lesions succeeded in upregulating the ipsilesional primary motor cortex and exhibited a functional improvement of upper limb motricity. However, its use in clinical practice seems limited due to technical constraints.

The other hybrid system in development is fNIRS-EEG. This system is mobile, making it easier to use in clinical practice. Although it was principally tested in healthy subjects, it could help to target and find better areas for the use of NFB in patients (Buccino et al., 2016).

Finally, it seems that the effects of unimodal versus hybrid acquisition systems are in favor of the hybrid approach (Buccino et al., 2016; Perronnet et al., 2017) and commercial hybrid systems are emerging (*G.TEC, Neurolite*). However, it should not be forgotten that their real impact is made at the price of a long technological investment and not necessarily easy to set up in research, and especially not in current care. Yet, these hybrid systems can bring the promise of a satisfying technological contribution in the future. The principal barriers on the rise of NFB related to the device are its access in the medical department, its cost, and the ability of a practitioner to use it daily. Therefore, to reach a common use, the acquisition systems must address the following issues: accessibility, comfort, effectiveness, accuracy, and adaptation to the clinical post-stroke context.

#### Different Approaches for Signal Processing

Another dimension to consider is the processing techniques for the EEG signal. Concerning MRI or NIRS acquisitions, data extraction is also a technical and engineering challenge that will not be discussed here, as EEG is currently the most common signal. The large variety of algorithms to process the raw EEG data leads to different results and may affect the quality of the biomarker recording. We suggest considering the pathophysiological characteristics of the stroke population to offer an adapted signal processing method that may lead to better rehabilitation techniques. As explained in the meta-analysis by Mansour et al. (2021), the recorded EEG raw signals must follow a pipeline in order to extract relevant information that can later be translated into feedback for the user. This pipeline involves three stages: preprocessing, feature extraction, and classification.

Typically, BCI systems are developed for a specific method, impeding homogenization and comparison of protocols. Some solutions have been developed to facilitate the implementation of BCI protocols, including neurofeedback. Some examples of these solutions are BCI2000 (Schalk et al., 2004), OpenViBE (Renard et al., 2010), NeuroPype (Intheon Labs, San Diego, CA, United States), Neuromore studio (Neuromore), BCILab (Kothe and Makeig, 2013), among others. These tools allow for recording brain signals from different acquisition systems, processing and using such signals for different ends such as controlling a robot, or neurofeedback. On the other hand, to perform offline analysis of EEG signals, one can use Matlab toolboxes such as EEGLAB (Delorme and Makeig, 2004), ERPLAB (Lopez-Calderon and Luck, 2014), and FieldTrip (Oostenveld et al., 2011) as well as the Python package MNE-Python (Gramfort, 2013), to mention a few tools.

The preprocessing stage aims at preparing the signal to facilitate the extraction of physiological characteristics. Here, we present some of the existing preprocessing approaches in a very condensed manner as they are out of the scope of this review, thus we strongly advise the reader to refer to the following reviews for more details on these techniques (Al-ani and Tr, 2010; Abuhashish et al., 2014; Lotte et al., 2018). They described three preprocessing steps: referencing, temporal filtering, and signal enhancement. Among the differences between studies, one of the most common is referencing. During EEG recordings, brain activity voltage is measured by a given electrode with reference to another one, commonly placed on the nose, earlobes, or mastoids. This is different from the computational reference, which may involve: common reference, average reference, current source density (CSD), rest, along with others (Al-ani and Tr, 2010; Lei and Liao, 2017). The relevance of re-referencing is to choose a reference where the electric potential is neutral or almost zero so that the measurement is free of contamination. Temporal filtering is used to remove artifacts caused by, for example, muscle activity, or any other noise from the environment. One of the most used techniques in BCIs for stroke rehabilitation is a band-pass filter between 8 and 30 Hz to extract relevant brain waves (Lotte et al., 2018). Lastly, signal enhancement is used to enhance the signal-to-noise ratio, it includes, but is not limited to spatial filters, principal component analysis (PCA), independent component analysis (ICA), common spatial patterns (CSP), common spatial subspace decomposition (CSSD), frequency normalization (Freq-Norm), among others. For more details on signal enhancement techniques used in BCIs, we refer the reader to Bashashati et al. (2007) and Al-ani and Tr (2010).

The feature extraction stage has the objective of detecting relevant and distinctive characteristics of biomarkers. In Mansour et al. (2021) meta-analysis, the most used features were band power (BP) features, common spatial pattern (CSP) features, and filter bank common spatial pattern (FBCSP) features. The group of studies that used band power features had the highest significant effect on motor function recovery. The authors suggest that in the long term the use of BP features potentially helps subjects to better modulate their brain patterns, leading to better motor recovery whereas the FBCSP features led to a better BCI-performance.

Choosing a classifier is not a trivial task either. This choice depends on a variety of factors such as the tasks, number of classes, the extracted features, the subject profile, among others. Due to the heterogeneity among studies, comparing the classifier’s performance and its efficiency in motor rehabilitation is highly challenging. An effort was made by Bashashati et al. (2015), where they used several standard datasets to compare, in a general framework, seven classifiers with two different feature extraction methods involving band power. Contrary to the popular belief of linear discriminant analysis (LDA) being the best classifier for sensorimotor BCIs, the authors suggested that for a given subject, the choice of a particular classifier relies on the feature extraction method. There has been a growing interest in deep learning methods for MI classification. A recent study (Avilov et al., 2020) tested three deep learning methods, including DeepConvNet, ShallowConvNet, and EEGNet, to detect MI with Median Nerve Stimulation (MNS) versus MNS during rest to prevent Accidental Awareness during General Anesthesia. The authors demonstrated that the deep learning network EEGNet outperformed not only traditional classifiers like CSP-LDA and the minimum distance to Riemannian mean algorithm (MDRM), but the other two deep learning architectures as well. However, further studies must be made using these methods for stroke participants, especially since a better performance does not necessarily implies a better post-stroke rehabilitation clinical outcome. For instance, whole-brain complex classification algorithms may end up classifying activity from electrodes not related to motor activity (such as prefrontal electrode activity), whereas more simple classifiers may focus on primary motor sensorimotor rhythm fluctuations. Hence, more studies on the comparison of the signal analysis process in BCIs-NFB for the post-stroke population are required to arrive at a conclusion for the selection of such algorithms according to the participant’s characteristics.

As previously stated, the principal biomarker used to detect movement-related patterns in the NFB is the ERD. However, new biomarkers are emerging, such as connectivity. They consider the brain’s ability to communicate at distance and functionally interact in a network (Mottaz et al., 2015; Gonzalez-Astudillo et al., 2021). One double-blind controlled study used it in post-stroke motor rehabilitation with a significant clinical result (Mottaz et al., 2018). However, in this study, subjects were not asked to perform MI techniques. Recent evidence suggests that it may be possible to use indices of functional connectivity using MI tasks (Cattai et al., 2021). More studies are needed to understand the differences observed.

A wide range of signal analysis tools is used in the studies and significantly change the types of analysis. One challenge would be to homogenize the computer processing of brain signals between studies, or at least to include some common conditions to develop the methodological robustness of the studies by examining the merits of promising pipelines in a larger clinical population. As mentioned before, another way to facilitate the comparison of signal processing techniques within protocols is to use the previously mentioned software dedicated to BCIs and allow a relatively easy and fast way to change these techniques while maintaining other aspects of the protocol intact. In addition, examining new pipelines in preliminary studies remains important to develop our knowledge. Then, the use of new biomarkers, classifier performance, and signal processing algorithms adapted to pathophysiological knowledge and brain function after a stroke is an appealing approach to the development of post-stroke NFB.

#### Feedback Modalities

Delivering feedback in BCIs and NFB therapies is relevant so that users may learn the task effectively and improve their performance (Jeunet et al., 2015a). These closed-loop interfaces are key for stroke rehabilitation by establishing a link between the desired movement and the body by generating proprioceptive activity in the paralyzed limb, stimulating neuroplasticity, and having an important effect on motor recovery (López-Larraz et al., 2018). Consequently, we explored the most common feedback modalities present in the literature. Unimodal feedback involves only one type of feedback related to a single sense only, such as visual or haptic modalities. Naturally, some multimodal approaches are starting to emerge, using two or more types of feedback, aiming for better and more intuitive control of the BCI (Gürkök and Nijholt, 2012), and potentially, an improvement in the rehabilitation process.

##### Visual Feedback

###### Standard Design

Visual feedback gives information to the users about their control performance through a graphical interface via a screen. Despite its simplicity, this type of feedback has not been broadly explored in control trials for stroke rehabilitation and has been further studied in combination with other modalities.

In Pichiorri et al. (2015), a study was conducted with 28 subacute stroke participants, giving visual feedback every time motor imagery was detected. The feedback consisted of a visual representation of the participant’s hands displayed on a white blanket that covered them. When MI activity was detected and it reached a predefined target, the virtual hands performed a grasp and finger extension movement. A feasibility study (Prasad et al., 2010), proposed a visual environment where five chronic stroke participants were meant to place a ball inside a basket by performing MI of the left or right hand. Despite the moderate BCI performance, participants showed an improvement in clinical rehabilitation measures. In Mihara et al. (2013), 10 post-stroke participants performed video-guided motor imagery of flexion and extension of the elbow as well as finger extension. Then, the video was replaced by a bar serving as NFB triggered by hemoglobin signals from a NIRS system. Ten more participants were allocated to a sham group, where the NFB was given randomly. The participants receiving the hemoglobin-triggered NFB presented greater improvement in the clinical measures as well as in the imagery-related cortical activation in the premotor area.

Another study (Rayegani et al., 2014) was conducted with 30 chronic-stroke participants where visual NFB was complemented with occupational therapy and compared to conventional therapy or electromyographic biofeedback associated with conventional therapy. In this case, the visual feedback was given by the means of a computer screen where a three-boat race was displayed. If the subject was able to keep sensorimotor rhythm (SMR) power above a predefined threshold and beta and theta waves power below another threshold, then the participant’s boat would advance further than the other two boats. The NFB and biofeedback therapies resulted in similar improvements in hand function to conventional therapy alone. However, the NFB modality showed an improvement in SMR power, suggesting the visual feedback helped the motor imagery task.

All these studies aimed at giving visual feedback, sometimes as straightforward as a moving bar, or as complex as virtual hands, providing either abstract or realistic feedback regarding the movement to be performed. A way to adapt this feedback to the participants’ needs is to take into consideration their preferences, for instance, preferred colors, or the participant’s physical characteristics to create a similar human avatar. With the rise of virtual reality, more complex and controlled environments have emerged, which provide better engagement than simple stimuli and better control over multisensory stimulation.

###### Virtual Reality-Based Feedback

Brain–Computer Interfaces, in combination with a virtual reality (VR) environment, also aim at promoting neuroplasticity and motor recovery by controlling virtual or real devices (Wen et al., 2021). Although the feedback in VR is visual, immersive VR (IVR) requires additional hardware in the form of a head-mounted device (HMD), which represents a challenge when coupling it with BCI technology. The VR HMD must be carefully positioned so that it does not interfere with the electrodes placed on the scalp. The system REINVENT in Vourvopoulos et al. (2019) is an example of this type of feedback. They used an Oculus Rift headset to show an arm movement by the means of IVR once MI was detected with an EEG. Besides proving the feasibility of VR-based BCIs for stroke participants, they showed an increase in the clinical assessment scores after using the device and verified users’ acceptability. This study demonstrated that IVR can be used in stroke participants, however, the costs and technical complexity of the system must be taken carefully into consideration. In addition, some people may report vertigo, nausea, and/or dizziness when using VR, and participants with visual impairments may not benefit from this solution.

Overall, VR allows offering more controlled environments and some of them closer to a real-life situation, which might result in an increased attachment to the therapy. Further studies are required to evaluate its therapeutic effects but also to verify the compatibility between the VR headsets and the signal acquisition systems, such as EEG headsets.

##### Haptic Feedback

The term “Haptic” refers to “sensory and/or motor activity of the skin, muscles, joints, and tendons” (ISO 9241–910:2011, 2011 244: 1). In Fleury et al. (2020), a classification of haptic interfaces used for BCIs was suggested based on two senses: (1) the tactile sense, which involves the mechanoreceptors found on the skin, and (2) the kinesthetic sense, associated with the receptors found in muscles, tendons, and joints. Until now, most controlled trials with stroke participants that involve haptic feedback have tested mainly two types of interfaces: FES and orthoses, including robots and exoskeletons. The profile of the post-stroke participant may influence the choice of the device that will deliver the haptic feedback.

###### Functional Electrical Stimulation

Functional electrical stimulation (FES), a subtype of neuromuscular electrical stimulation (NMES), uses surface electrodes to deliver electrical stimulation. In this way, one can artificially induce functional muscle movement that might be used for daily-life activities (Popović, 2014; Marquez-Chin and Popovic, 2020). The intensity of such stimulation can be varied to determine the level of contraction and the muscles involved. This includes sensory threshold FES (Corbet et al., 2018), a type of electrical stimulation that will not induce movement, but its effects on stroke participants are yet to be explored.

This type of feedback can be used for the motor recovery of lower or upper limbs. In the work presented in Chung et al. (2020), a BCI using FES feedback was tested to evaluate its effects on postural control and gait performance in participants with hemiparetic chronic stroke. The stimulation was delivered in the tibialis anterior, with or without the use of a BCI. Gait velocity, cadence, and step length increased significantly after the BCI-FES training compared to the FES-only training, suggesting the system has potential advantages for stroke hemiparetic participants.

Functional electrical stimulation has also been used for upper limb rehabilitation involving reaching and grasping movements. In Ibáñez et al. (2017) a BCI was tested where FES was triggered once reaching movement intention was detected by EEG signals. The clinical assessment (FMA-UE and stroke impact scale) of four stroke participants improved significantly after using the system, and the participants’ assessment showed their acceptance of the solution. The protocol in Biasiucci et al. (2018) aimed to help recover the hand extensors by triggering the FES every time MI was detected. A control group of 13 stroke participants had FES triggered independently of the brain activity, while the BCI-FES group of 14 stroke participants activated the system according to the MI activity. Contrary to the control group, the BCI-FES group showed significant functional motor recovery.

Functional electrical stimulation feedback can be used in other contexts than reaching and grasping. A randomized controlled trial was presented in Jang et al. (2016) to treat shoulder subluxation. Both the BCI-FES and FES-only groups consisted of 10 stroke participants. The BCI-FES group had the FES system triggered when EEG patterns corresponding to shoulder motions were detected. The participants in this group were watching a video of the actions to perform and would then attempt to perform them by themselves. After using the BCI-FES system, four scores of clinical assessments improved, while only three scores of the FES-only group improved. Their results suggest this training may help in the motor recovery of the shoulder in stroke participants.

Additionally, FES can be complemented with action observational methods like in Kim et al. (2016) where 30 chronic-stroke participants observed videos of daily life tasks. Fifteen participants had an EEG system that was used to measure attention level and motor imagery. Whenever these two reached a defined threshold, FES was triggered to stimulate the extensors of the wrist. Their results showed a significant increment in clinical assessments of the participants using the BCI-FES system versus the 15 participants in the control group following conventional therapy.

Functional electrical stimulation feedback systems have proved to be relevant for the motor recovery of stroke participants. Nevertheless, they present some limitations such as the need for specific devices that also require specialized personnel for operation, and the limb muscles should be available, which could be a problem when spasticity is present.

###### Orthoses and Robots

Orthoses, such as exoskeletons, and robots are being used to help the participants perform a specific movement. In this context, most of the controlled groups are presented with sham feedback where the exoskeleton movement is independent of the MI task.

Exoskeletons allow assisting the participant in performing the movement and to give some proprioceptive feedback when coupled to a BCI. Indeed, most of the developed devices trigger the finger extension once MI has been detected, as most stroke participants present spasticity in the form of a closed hand. One study (Ramos-Murguialday et al., 2013) involving 32 post-stroke subjects, studied the effects of an orthosis that performed reaching and grasp movements that were either dependent on the brain activity or randomly delivered. A larger increase of the clinical assessment (FMA) was observed on the subjects that followed the NFB protocol rather than the control group. In Bundy et al. (2017), a powered exoskeleton opened and closed the hemiparetic hand of ten chronic stroke participants. The NFB training was done at the participant’s home for 12 weeks. An increase in the primary clinical assessment (ARAT) showed the feasibility of successfully performing NFB training at home with an exoskeleton. Lastly, a multicenter study consisting of 74 post-stroke participants, showed a larger increase in the FMA and ARAT scores of the BCI-controlled exoskeleton group than the control group.

Robots also aid with movement execution after MI. An example can be found in Bhagat et al. (2020), where they used the MAHI EXO-II device to help with elbow motor recovery. The robot would trigger whenever a motor attempt was detected with EEG and corroborated with EMG. No sham feedback was presented, however, the participants that followed the protocol presented significant improvements in arm/hand movements and coordination but not in hand strength and velocity, which was expected as the robot focuses on the elbow. On the other hand, in the study presented in Várkuti et al. (2013), a Manus robot, used for shoulder and elbow recovery, was activated whenever MI was detected or 2 s after no activity was detected. Their results showed an improvement in clinical scores and in functional connectivity, which was measured by resting-state fMRI.

One difference between exoskeletons and robots is their portability. While most robots are grounded to the floor or a base, and cannot be taken out of the hospital environment, some exoskeletons are portable. In this case, the participants may take the device to their home and follow the rehabilitation process in a more comfortable and familiar environment, which may increase acceptability and adherence. In addition, the therapists or any other qualified personnel do not have to be always present with the subject, allowing them to take care of more participants at the time. An example of a portable BCI-exoskeleton device is the IpsiHand by Neurolutions, recently approved by the Food and Drug Administration of the United States of America.

These types of devices should be conceived so that they are adaptable to the morphology of the participants, especially for those who present spasticity. Moreover, some of them (like hand exoskeletons) may be adapted to the environment, whether it is at home or the hospital.

Haptic devices allow greater interactivity of the stroke subjects with the BCI by providing sensorimotor feedback. In this way, the subjects may understand better how to modulate their brain signals, which may result in better recovery. Nevertheless, these devices must be adapted to the limitations of the participant, physical and cognitive, to their environment, and to their rehabilitation needs.

##### Multimodal Feedback

Multimodal feedback in BCI is described as a type of feedback where two or more sensory modalities are stimulated as a response to brain activity. Most efforts have focused on combining visual feedback with a haptic one, as it is expected to deliver a better experience to the user and result in improved performance (Gürkök and Nijholt, 2012). Naturally, one can say that feedback resulting in movement, such as FES, robots, and exoskeletons, combine haptic and visual modalities. Because they reproduce movement, the participant can feel and see their limb moving. One way to isolate this feedback would be to cover the limb so that the participant cannot see it. However, most studies have not made this differentiation, making it hard to evaluate the effects of these visual stimuli.

Future perspectives may combine other types of feedback. An example can be visual feedback with a vibrotactile one, as in Leeb et al. (2013), Jeunet et al. (2015b), and Pillette et al. (2021), yet, it has to be tested in the stroke population. This type of feedback has proven to liberate the visual channel so that subjects can pay attention to other events such as distractors (Leeb et al., 2013; Jeunet et al., 2015b). It could also be complemented with robots, exoskeletons, or auditory feedback.

As we have seen, the feedback modality in the presented studies has been independent of the participants’ profiles. Here again, the diversity of solutions opens up possibilities for adaptation to the singularity of patients. Indeed, the feedback can be informational (score, performance gauge, boat race, etc.), more embodied (first-person view of virtual hands, haptic feedback, etc.), or even assistive devices (robotics, orthoses). However, homogenization of the participants’ profiles and in the protocols should be done so that studies may be compared. Questions also remain to be answered regarding the possible mental load or dissociation of attention in the face of multimodality for patients who are tired and have a reduced attentional capacity. In particular, the synchronization or not of feedback is also a track to explore. An additional dimension to be evaluated is the effect of the feedback on the participants’ motivation and overall user experience, which is commonly overlooked. It is possible that the personal emotional and pragmatic experiences of the participants have an important role in the NFB training outcome, thus they should be taken into consideration. Depending on the feedback, we can choose to involve different neural loops and brain circuits according to the lesions and the rehabilitation aims.

## Discussion

### Summary of Feedback, Clinical Context, and Signal Acquisition System

We summarized the different feedback modalities presented in the literature, along with the time elapsed since the stroke, lesion location, type of stroke, measurements employed for the clinical assessment, and the signal acquisition system to offer a concise comparison between studies (Table 2). While all studies indicate the time since stroke, this is not the case for the stroke type: ischemic or hemorrhagic, nor for the lesion site, which are often not mentioned. This missing information represents a comparison obstacle to propose feedback according to the participant’s characteristics associated with the stroke.

**TABLE 2:** Summary of types of feedback versus the time since stroke, lesion location, type of stroke, measurements employed for the clinical assessment, signal acquisition system, and features used for classifications.

Type of feedback	Study	Time since stroke			Lesion location	Type of stroke		Measurements for clinical assessment	Signal acquisition system	Features
		*Chronic*	*Acute*	*Subacute*		Ischemic	Hemorrhagic			
Visual	Pichiorri et al., 2015			✓	Unilateral, cortical, subcortical, or mixed stroke	✓	✓	FMA-UE, MRC, NIHSS, upper limb section of the MAS for spasticity	EEG	Band Power
	Prasad et al., 2010	✓			NI	NI	NI	ARAT, NHPT, GS, McI	EEG	Band Power
	Mihara et al., 2013			✓	Subcortical (Putamen, Corona radiata)	✓	✓	FMA-UE, ARAT, MAL, KVIQ-10	NIRS	Band Power
	Rayegani et al., 2014	✓			NI	NI	NI	JHFT	EEG	Band Power
Immersive Virtual Reality	Vourvopoulos et al., 2019	✓			Subcortical	NI	NI	FMA-UE, MAS, SIS	EEG	Band Power
Functional Electrical Stimulation	Chung et al., 2020	✓			NI	✓	✓	TUG, BBS	EEG	Band Power
	Ibáñez et al., 2017	✓			Middle cerebral artery	✓	✓	FMA-UE, Stroke Impact Scale	EEG	Band Power
	Jang et al., 2016	✓			NI	NI	NI	Shoulder subluxation: vertical distance, horizontal distance. Pain: visual analogue scale (VAS). Manual Function Test (MFT)	EEG	Band Power
	Kim et al., 2016	✓			NI	✓	✓	FMA-UE, MAL, MBI, ROM of paretic arm	EEG	Band Power
	Biasiucci et al., 2018	✓			Subcortical, Cortical	✓	✓	FMA-UE, MRC	EEG	Band Power
Exoskeleton	Bundy et al., 2017	✓			Subcortical, Cortical	✓	✓	ARAT, MAS	EEG	Band Power
	Ramos-Murguialday et al., 2013	✓			Subcortical, Cortical	NI	NI	FMA-UE	EEG	Band Power
Robot	Várkuti et al., 2013	✓		✓	NI	✓	✓	FMA-UE	EEG	Band Power
	Bhagat et al., 2020	✓			Subcortical, Cortical	✓	✓	FMA-UE, ARAT, JHFT, pinch and grip strengths	EEG	Band Power

*NI, no information was provided by the authors; FMA-UE, Fugl-Meyer Assessment for upper extremity; MRC, Medical Research Council scale for muscle strength; NIHSS, National Institute of Health Stroke Scale; MAS, Modified Ashworth Scale; ARAT, Action Research Arm Test; NHPT, Nine Hole Peg Test; GS, grip strength; McI, upper limb movement and motor control: Motricity Index; MAL, Motor Activity Log; KVIQ, Kinesthetic and Visual Imagery Questionnaire; JHFT, Jebsen Hand Function Test; TUG, Timed Up-and-Go test; BBS, Berg Balance Scale; MBI, Modified Barthel Index; ROM, Range Of Motion; SIS, Stroke Impact Scale.*

Until now, it appears the type of stroke, whether ischemic or hemorrhagic, does not influence the choice of the feedback modality. Similarly, the type and severity of the participant’s deficit are often not taken into consideration for the type of feedback. In addition, some authors prefer to include participants with subcortical lesions as there is no damage on the cortex and participants are less likely to present cognition impairments. Most of the studies have been conducted with chronic participants, while just a few have been conducted in the subacute population, and none in acute participants. Once the participants have been stabilized and the motor impairments have been defined, the rehabilitation process may start, which is most common during the chronic phase.

### Neurofeedback Limitations

#### Study Protocols Limitations

For over a decade, numerous studies have focused on the NFB for post-stroke rehabilitation but with heterogeneous protocols, which causes several problems to study their efficacy. This heterogeneity may lead to significant confusion in the analysis of these studies, with biases and difficulties in a systematic assessment.

One of the many limitations of the advancement of understanding in the field of neurofeedback is probably related to biases in the literature. The majority of publications report studies with positive results and not the protocols that did not succeed. This bias distorts the results of meta-analyses and systematic reviews, and should lead to caution in the interpretation of results. Although negative, these studies would allow us to better understand the mechanisms, and would bring us larger cohorts.

##### Heterogeneity in the Conduct of the Protocol

The duration and time of the NFB therapies vary widely throughout the protocols. Bai’s meta-analysis showed the diversity of the protocols in 14 randomized and controlled studies (Bai et al., 2020). They included an average of 15 sessions (from 6 to 30 sessions, 2 to 5 times a week, 30 min to 1 h30 per session) with a total average of 12 h of NFB therapy (from 1 to 24 h in total). The duration and the repetition of the motor imagery (MI) trials also varied across the different studies: from 4 to 18 s. In general, clinical and cortical significant results were highlighted in protocols performing several hours (more than 10 h) of NFB (Ramos-Murguialday et al., 2013).

We noticed a high heterogeneity in the control groups of the studies. For instance, in studies where a FES system was evaluated alongside NFB, the control groups vary among each other. In some studies, the control group will consist of a FES-only system (Jang et al., 2016) while in other studies the control group concerns only the BCI without any feedback. Moreover, in Kim et al. (2016), the effects of a BCI-FES were compared to conventional therapy. This difference in protocols may represent a problem when trying to compare and evaluate their efficiencies. Therefore, we suggest evaluating the following conditions whenever possible, in addition to conventional rehabilitation: conventional therapy only, brain modulation without any feedback, sham feedback (i.e., feedback independent of the brain activity), and finally NFB with sensorial feedback, which is a closed-loop BCI.

##### Clinical Ability to Practice Neurofeedback

Lastly, an important limitation of these studies is the type of brain injury acquired and the resulting sequelae (Broussy et al., 2019). Theoretically, the goal of NFB is to modulate motor areas related to motor deficits. However, in many cases, the lesions also affect other areas resulting in combined impairments of sensitivity, vision, language, and cognition, which may alter the ability to practice NFB. Therefore, most studies exclude the subjects with these disorders from their cohort, for they are judged to be troublesome for the correct application of the protocol. This choice leads to two limitations: a lack of representation of the general post-stroke population who often have several clinical impairments and not only motor ones, and a challenge in establishing the threshold level of symptoms that may interfere with performance in NFB.

##### Heterogeneity in the Upper Limb Evaluation

Most studies in NFB post-stroke motor rehabilitation focus on upper limb rehabilitation because its deficiency remains a major issue after stroke (Nakayama et al., 1994). The majority of studies used common motor upper limb scales such as FMA-UE, ARAT, which is in line with a meta-analysis on the outcome measures of stroke rehabilitation studies on the upper limb (Santisteban et al., 2016). Other studies used functional scales such as MAL, BBS, or global motor scales such as MRC and NIHSS (Table 2). This diversity in the pre and post-intervention assessments increases the difficulty of comparing them. Nevertheless, most of them highlight significant positive differences after NFB training on the clinical scales used which globally demonstrates the efficacy of the protocols. Multiple reviews and meta-analyses have shown these results (López-Larraz et al., 2018; Carvalho et al., 2019; Bai et al., 2020; Kruse et al., 2020). Another limit concerning these scales is their ceiling effect that can mask the evolution of the subjects.

##### Evaluation of the User’s Experience

To perform a coherent and useful NFB protocol, we also need to know if the studies satisfy the participants and meet their expectations. Indeed, models of care are shifting from a disease-centered model to a more complex human-centered model (Graffigna et al., 2013). Thus, the increased demand for wellness leads to questioning the boundaries between care and treatment, and to propose so-called positive technologies (i.e., that provide positive personal experiences by stimulating positive emotions, a form of connectivity between humans, caregiver-patient for example, an acceptance and engagement in the care pathway). However, few studies focused on the user’s sensation and experience after using the devices (Chowdhury et al., 2018; Foong et al., 2020). Most studies tried to test the feasibility of using new devices (EEG cap, acquisition system, ease of set-up) with encouraging results (Nishimoto et al., 2018; Jochumsen et al., 2020).

##### Accessibility to Neurofeedback Training on an Outpatient Basis

We must consider the possibility of accessing NFB training according to the subject’s environment. Most studies performed the NFB training at the hospital on an outpatient basis when the participants were in a better general condition but still suffered motor deficiency and needed rehabilitation. At the early stage of stroke, few studies have used portable devices to practice NFB training at the patient’s bedside (Hashimoto et al., 2021). Two studies focused on testing the devices at the subject’s home. In order to allow better compliance with a complete four weeks NFB program for three chronic post-stroke participants, one study provided home-based practice to follow NFB training, with cortical changes highlighted for these subjects (Zich et al., 2017). Another study used exoskeleton feedback to provide proprioceptive stimulation in a 12 weeks program for 10 chronic stroke subjects, with a statistical improvement in upper limb motor function and proof of technical feasibility at home (Bundy et al., 2017). Portability allows the participants to take the device with themselves, which may increase the time dedicated to the rehabilitation process, as well as comfort by using it at home.

To summarize, heterogeneity in NFB protocols does not currently allow a robust comparison of parameters. Moreover, new settings are to be taken into account such as user experience, portability and accessibility in daily practice.

#### Feedback Limitations

We offer a general overview of the clinical limitations for specific feedback, providing a glance at the main points to consider (Table 3). These limitations include especially aphasia, ataxia, spasticity, hemiparesis, skin injuries and/or diseases, metal implants, pacemakers, among others. However, other dimensions are often not taken into consideration such as the need for experienced operators, adaptability of the device, and the financial investment.

**TABLE 3:** Properties to take into consideration while selecting the feedback nature for a post-stroke subject.

	Visual	Virtual reality		Haptic		
		*Immersive*	*Non-immersive*	*Robots*	*Exoskeletons/Orthoses*	*FES*
**Solution Characteristics**						
Portability	Possible	Possible	Possible	Not possible	Possible	Possible
Adaptability	Yes/High	Yes/High	Yes/High	Low	Medium	Yes/High
Experienced operator	Not needed	Maybe	Not indispensable	Yes	Depends on the complexity of the device	Yes
Financial Investment	Medium	High	Medium	High	High, some low-cost approaches possible	High
**Clinical Limitations**						
Spasticity				Might affect	Depends on severity	Might affect
Severe aphasia	–	–	–	–	–	–
Severe ataxia	Might affect		Might affect	–	–	–
Metal implants						–
Pacemakers						–
Skin injuries or diseases					Might affect	–
Visual impairment	–	–	–			
Seizures	–	–	–	–	–	–
Severe hemineglect	–	–	–	–	–	–
Severe arthritis	–			–	–	
Cognitive Impairment	–	–	–	–	–	–

*The “–” symbol means that the presence of the clinical limitation minimizes the possibility of using the feedback modality.*

The need for an experienced operator is relevant to consider as it will affect other dimensions, like portability and financial cost. When certain feedback requires a specialist, such as a technician or a physical therapist, the use of the device will be most likely constrained to a clinical environment and naturally, its overall cost will increase. Indeed, the financial investment dimension can be linked to the feedback device itself, as well as other costs like therapists, technicians, and/or hospital or rehabilitation center fees. In addition, the financial cost may increase proportionally to the complexity of the technology involved. For example, an EEG-compatible headset is needed for immersive VR, increasing the cost, while visual feedback using just a screen might be more financially accessible. On the other hand, a device that can be operated by the subject or a relative increases the possibility of taking the device to the patient’s home, but this requires that the patient accepts and engages in their rehabilitation.

### General Recommendations for an Adapted Neurofeedback for Stroke

After reviewing the extensive literature on NFB therapy for post-stroke motor rehabilitation, we concluded that providing guidelines for an adapted therapy is premature as there are still plenty of questions to be solved regarding the different aspects we have previously presented, such as the anatomical targets to mention one. Nevertheless, we suggest some recommendations of the most important aspects to be considered when selecting the type of feedback modality. Alternatively, we offer research perspectives that will allow the scientific community to find answers to the questions that will allow us to offer an adapted NFB therapy for the post-stroke population.

In Table 3, we have summarized the different properties to consider while choosing a type of feedback. We suggest involving frequently overlooked aspects such as the device’s portability, adaptability to the participants and thus their environment, the financial investment, and the possible need of an experienced operator to use the device. It is important to note that these dimensions are in constant interaction with each other thus changing one or a few of them will impact the others.

We also suggest developing feedback that may be adjusted to the participant’s limitations and characteristics, for example, adapting to the physical constraints of the participant, like difficult access to the limb due to spasticity, or personalizing an avatar to resemble the participant’s real limb. Finally, another key property includes clinical limitations. Most studies focus on selecting the participants according to the feedback modality. However, we propose an approach that recommends the feedback according to the participant’s needs and limitations. For this reason, it is important to consider the clinical limitations of each type of feedback.

### Perspectives

By summarizing the work on NFB therapies in post-stroke motor rehabilitation, we highlighted the difficulties of improving the techniques in light of current knowledge and we have talked about the use of NFB in a real clinical context. The first point to further discuss is the lack of homogeneity of the studies, preventing the data comparison. In Jeunet et al. (2018), the author already suggested that the technical parameters used in NFB must be adapted. More recently, an interesting review tried to make recommendations and further work to improve NFB protocols, focusing on somatosensory impairments as an important limit in this therapy (Pillette et al., 2020). In this review, we expand this point of view by considering all the settings individually which constitute the studies in NFB and proposing new research perspectives in the light of recent developments. The main strategy to adopt is first to homogenize the studies and test these different parameters to better understand their indication. Even if the suggestion of adapting the NFB to the clinical context seems paradoxical, it is crucial to progress in the way of adapting to the needs of patients in their heterogeneity. From these different data, adaptive protocols may emerge. For example, details on the type of stroke (affected brain structures, localization), lateralization, clinical severity at the onset, cognitive state, and neurological history may be useful. It is recommended for future studies, whether they are feasibility studies or controlled randomized trials, to report homogenized information about the clinical context so that further analysis may be done on these dimensions and more adapted NFB therapies may be offered to the participants.

We focused in this paper on the motor rehabilitation after stroke, but interesting literature has paved the way for a more general and comprehensive use of NFB in post-stroke motor, cognitive and emotional rehabilitation (Mane et al., 2020). Indeed, other impairments such as cognitive dysfunction and emotional impairment are common post-stroke disorders that affect patients’ quality of life and access to rehabilitation. In the literature, attentional disorders after stroke were observed in 50% of cases, language disorders in 30% of cases, mood disorders in 60% of cases (Parikh et al., 1990; Leśniak et al., 2008; Jokinen et al., 2015; Flowers et al., 2016). These disorders were more recently targeted by NFB. Preliminary studies have focused on the use of NFB with alpha wave regulation in post-stroke cognitive and emotional disorders with encouraging results (Kober et al., 2013; Toppi et al., 2014; Kübler et al., 2017; Saj et al., 2021). Considering these deficiencies and their impact on the use of NFB for motor rehabilitation, it appears essential to find therapeutic solutions adapted to these very frequent multi-deficiency situations.

**FIGURE 4:**
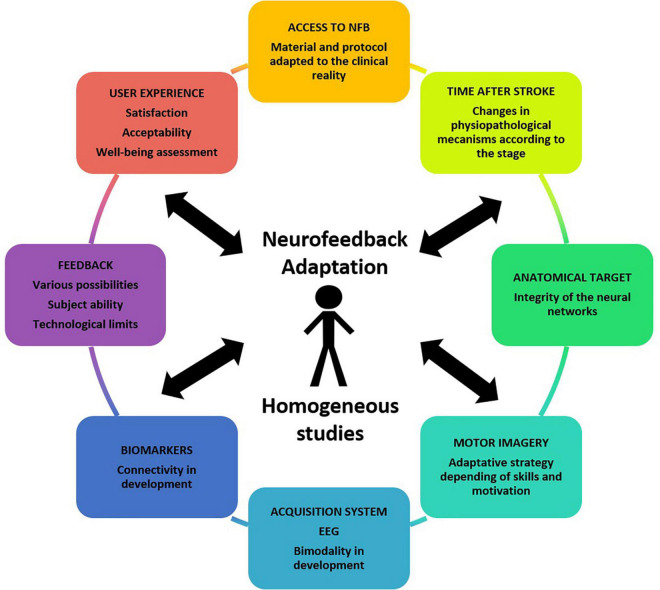
Clinical, electrophysiological, pathophysiological, and material parameters to be taken into account in an NFB study.

To improve the state of research, we must take into consideration the global situation of the participants, including their clinical context and the technical parameters to adapt. Several lines of research in this area are, in our opinion, possible directions for adapted NFB (Figure 4):

❖ The modalities of NFB according to the post-stroke time (Section “Time-based target”);
❖ The distinction of the type of NFB according to the lesion (subcortical, cortical, size and impact of adjacent anatomical structures, network) (Section “Choice of brain biomarkers for enhancing motor recovery”);
❖ The weighting of stimulation according to the localization, the motor skills to be rehabilitated, evolving in the course of time after stroke (Sections “Time-based target” and “Choice of brain biomarkers for enhancing motor recovery”);
❖ A more precise understanding of the motor imagery process in post-stroke subjects (Sections “Motor Imagery or Motor Attempt” and “User’s disability in BCI”);
❖ Equipment and protocols adapted to the clinical reality of stroke subjects, to their physical and cognitive deficits, accessible to the practitioner and the subjects. Systems accessible for inpatient or at home, convenient for daily use (Section “Summary of feedback, clinical context, and signal acquisition system”);
❖ The search for new locations to target NFB or new biomarkers. This takes better account of the motor area network (Section “Neurofeedback Limitations”);
❖ Automated systems but adapted to the patient’s clinical evolution (Sections “Summary of feedback, clinical context, and signal acquisition system” and “Neurofeedback Limitations”);
❖ Feedback adapted to the clinical deficit and identification of modalities for potentiating neuromodulation (Section “General Recommendations for an adapted Neurofeedback for stroke”);
❖ Evaluation of the acceptability, user experience, satisfaction and well-being during the protocols by the patient and the therapist, which is currently almost non-existent (Section “Evaluation of the User’s Experience”);

In this review, we were interested in the adaptation of the NFB to a post-stroke population, taking into account the maximum number of criteria and thus we considered research perspectives (Figure 4). most advanced proposal would be an adaptation of all the parameters to the subject, perfectly suited to his/her needs to obtain the most effective treatment and taking into consideration his/her personal preferences. This concept is future-oriented but highlights the need to take into account all the parameters, including at the individual level, as explained in this review to enhance the therapies. In this last perspective, we would no longer speak of “adaptation” but of “personalization.” As explained in Biro’s study, “Personalized medicine refers to a medical model using molecular profiling for tailoring the right therapeutic strategy for the right person at the right time” (Bíró et al., 2018). The best rehabilitation would be an active search for the most effective solution for an individual.

## Conclusion

We have scanned the different parameters constituting an adapted NFB for post-stroke recovery. Although guidelines are hard to establish, we have summarized the possibility of selecting each important factor to build a NFB protocol adapted to the post-stroke subject and the technical requirements. Some parameters can already be partly adapted to the clinical deficit of post-stroke subjects, such as the types of feedback. Other parameters are being explored: bimodality of the acquisition systems, other motor pathway targets, target adaptation over time, among others.

A better understanding of the physiopathological phenomena after a stroke based on modern neuroscience and larger homogeneity of studies and protocols will allow us to circumscribe the field of use of NFC better. Several lines of research remain to be developed to optimize this treatment method. NFB techniques remain innovative approaches and their common use practice is far from widespread. Therefore, new approaches, as close as possible to post-stroke subjects, must be considered.

We developed here an overall thinking including the clinical context surrounding the patient and the technological considerations from BCI literature. This original work has repositioned the use of NFB in a real clinical context and is trying to advance in the reflection of brain modulation strategies for the treatment of post-stroke sequelae. We need to adapt the methodology used in the protocols as close as possible to the needs of the target populations. Therefore, these protocols need to be as descriptive and transparent as possible to better understand their application and compare them to allow for evidence-based adaptation of NFB therapy. Indeed, the success of a method actually depends on multiple parameters. The challenge will be to obtain a system that can be widely used while remaining acceptable, accurate, and adaptive to be as efficient as possible. NFB remains an open door for future research.

## Author Contributions

SL, GH, and MG: conceptualization, methodology, and writing—original draft and review. SB: writing—review. SF and LB: writing—review and supervision. AL and IB: conceptualization, writing—review, and supervision. All authors: contributed to the article and approved the submitted version.

## Conflict of Interest

The authors declare that the research was conducted in the absence of any commercial or financial relationships that could be construed as a potential conflict of interest.

## Publisher’s Note

All claims expressed in this article are solely those of the authors and do not necessarily represent those of their affiliated organizations, or those of the publisher, the editors and the reviewers. Any product that may be evaluated in this article, or claim that may be made by its manufacturer, is not guaranteed or endorsed by the publisher.
